# Bossanger’s Catalogue of Anatomy

**Published:** 1871-08

**Authors:** 


*Bossange's Catalogue of Anatomy.*

This Catalogue contains a complete list of illustrative preparations of a complete course of internal and external pathology of and descriptive anatomy and surgery.

